# Association between cardiometabolic index and primary hypothyroidism: A cross-sectional study based the NHANES (2007–2012)

**DOI:** 10.1097/MD.0000000000049270

**Published:** 2026-06-12

**Authors:** Xue-kang Ren, Yu Fu, Shao-gong Zhu, Rong-zhen Li, Yun-zhan Xia

**Affiliations:** a Department of General Surgery, The Fifth Clinical Medical College of Henan University of Traditional Chinese Medicine, Henan Zhengzhou, China; b Department of Neurology, The Fifth Affiliated Hospital of Zhengzhou University, Henan Zhengzhou, China.

**Keywords:** Cardiometabolic Index, cross-sectional study, NHANES, primary hypothyroidism, TSH

## Abstract

The Cardiometabolic Index (CMI) is emerging as a novel marker for assessing visceral obesity and dyslipidemia. This study aims to investigate the relationship between CMI and primary hypothyroidism among US adults. This cross-sectional study was conducted among adults with complete records of CMI and thyroid function information from the National Health and Nutrition Examination Survey (NHANES) 2007–2012. The calculation formula for CMI is Triglycerides (TG)/High-density lipoprotein cholesterol (HDL-C) × WHtR (WHtR = waist circumference/height). Weighted linear regression models and multivariate logistic regression models were employed to investigate the association between CMI and serum thyroid-stimulating hormone (TSH) level/primary hypothyroidism, respectively; additionally, restricted cubic spline (RCS) analysis was used to verify the nonlinear relationship. Subgroup analyses were performed to explore potential effect modifiers. 3304 participants, aged 20 years and older, were included from NHANES across 3 survey cycles. In this study, 6.69% of the participants were diagnosed with primary hypothyroidism. A higher CMI correlated consistently with increased serum TSH levels (β: 2.47; 95% confidence interval [CI]: 2.38–2.57) and primary hypothyroidism (odds ratio [OR]: 8.70; 95% CI: 4.93–15.37). In the fully corrected model, for each unit increase in CMI, the serum TSH levels increased by 0.70 mIU/L. Subgroup analyses showed that the association of CMI with primary hypothyroidism was stably present in all subgroups. Interaction effects were observed for gender and body mass index subgroup (*P* for interaction: < .05). RCS regression highlighted a significant positive nonlinear association between CMI and TSH level/primary hypothyroidism (*P* for non-linearity < .001). Our results indicated a significant positive association between CMI levels and serum TSH level and primary hypothyroidism risk among US adults.

## 1. Introduction

Primary hypothyroidism is a common global disorder characterized by thyroid hormone deficiency, with a prevalence rate of approximately 5%.^[1]^ It affects nearly all body systems, presents with a wide range of clinical manifestations-from asymptomatic cases to rare life-threatening ones, and is often closely associated with elevated thyroid-stimulating hormone (TSH) levels.^[2]^ Previous studies have shown that TSH plays an important role in regulating lipid metabolism, and elevated TSH concentrations are closely associated with a variety of lipid metabolism-related diseases, such as nonalcoholic fatty liver disease, cardiovascular disease, and are also associated with an increased risk of ischemic heart disease and heart failure.^[3,4]^ Risk factors for hypothyroidism include insulin resistance (IR),^[5]^ obesity,^[6,7]^ hyperlipidemia,^[8,9]^ and atherosclerosis,^[10]^ among which obesity is considered a particularly important factor. In morbidly obese patients, TSH is moderately increased, and weight loss provokes a diminution of the elevated thyrotropin values.^[11]^ This evidence suggests that obesity and the lipid metabolic state have an impact on TSH levels. Therefore, recommendations for the management of hypothyroidism usually emphasize the role of weight loss. Despite the well-established association between hypothyroidism and obesity, the metabolism-related indicators that can predict elevated TSH levels and primary hypothyroidism remain unclear at present. Therefore, finding an index that can predict serum TSH levels and primary hypothyroidism is essential to reduce the risk of primary hypothyroidism and improve the quality of life of patients.

The cardiometabolic index (CMI) is a novel adiposity index that integrates obesity-related parameters and lipid metabolism markers to evaluate the distribution characteristics and functional impairments of visceral adipose tissue.^[12]^ Compared with conventional lipid metabolism-related indicators, the CMI offers a more holistic assessment of an individual’s metabolic health status by integrating the waist-to-height ratio (WHtR) and triglyceride-to-high-density lipoprotein cholesterol ratio (TG/HDL-C). The WHtR has been recognized as a more accurate indicator for assessing specific health risks compared with body mass index (BMI), primarily attributed to its ability to characterize the distribution pattern of adipose tissue.^[13]^ Additionally, the TG/HDL-C ratio has emerged as a widely recognized biomarker for identifying lipid metabolism disorders.^[14]^ Studies have demonstrated that the CMI exhibits superior performance to BMI and single lipid parameters in predicting conditions including metabolic syndrome, gallstones, insulin resistance, and atherosclerosis.^[15,16]^ Therefore, CMI may be more suitable for assessing the risk of hypothyroidism than traditional indicators of obesity or lipids.

To our knowledge, the relationship between CMI level and primary hypothyroidism risk is still unclear. Hence, the objective of this study was to investigate the link between CMI and TSH level/primary hypothyroidism using the cross-sectional data from the National Health and Nutrition Examination Survey (NHANES) database to reveal the potential role of lipid metabolism in primary hypothyroidism.

## 2. Methods

### 2.1. Study aims

Primary aim: To investigate the independent association between CMI and serum TSH level as well as the risk of primary hypothyroidism among U.S. adults. Secondary aims: to explore the nonlinear and dose-response relationship between CMI and primary hypothyroidism, and to verify the stability of this association through subgroup and multivariable analyses.

### 2.2. Study population

This study employed data from 3 consecutive survey cycles conducted between 2007 and 2012, including all NHANES database records with reported Thyroid Profile. The NHANES is a nationally representative cross-sectional study conducted by the National Center for Health Statistics (NCHS) with a focus on assessing the health and nutritional status of the United States population.^[17]^ It covers a broad spectrum of topics, ranging from demographic data, socioeconomic indicators, dietary patterns, to various health parameters, and employs rigorous methodologies for data collection, including medical evaluation, physiological assessments, and laboratory analyses carried out by skilled healthcare professionals. The primary objective of the survey is to elucidate the prevalence and determinants of key health issues in the U.S. population and furnish evidence-based support for public health policy decisions. For more detailed information, kindly refer to the official website at https://www.cdc.gov/nchs/nhanes/?CDC_AAref_Val=https://www.cdc.gov/nchs/nhanes/index.htm.

**Figure 1.**
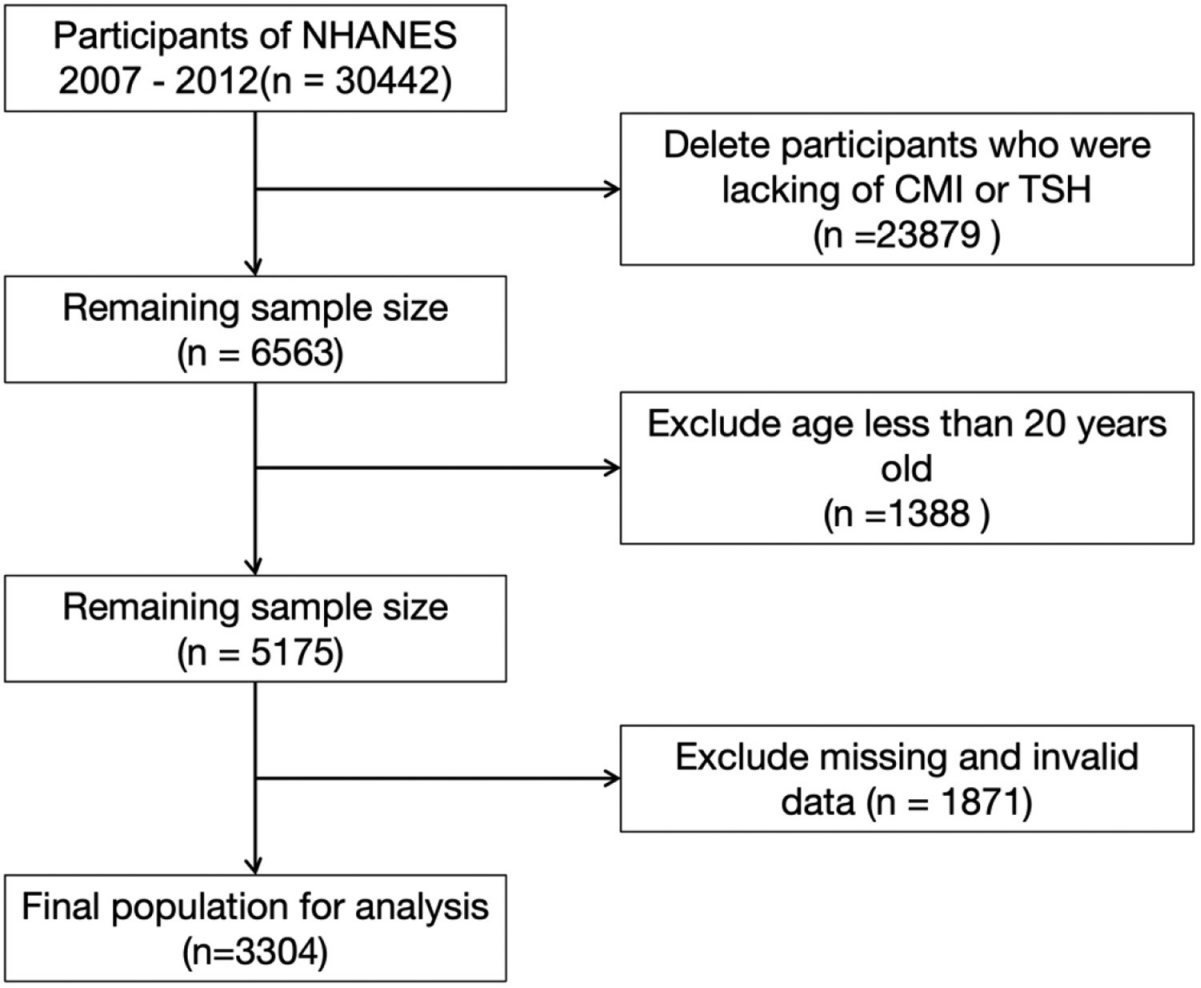
NHANES 2007–2012 participant selection flowchart. NHANES = National Health and Nutrition Examination Survey, CMI = Cardiometabolic Index, TSH = thyroid-stimulating hormone.

This study encompassed all participants from the 2007 to 2012 period, amounting to a total of 30,442 individuals. The participants included in this analysis possessed comprehensive demographic information, standard anthropometric measurements, lipid profiles, as well as details regarding lifestyle habits and medical conditions. The exclusion criteria were as follows: age < 20 years; lack of TSH or data for calculating CMI; missing covariate data, including race, poverty income ratio (PIR), education level, marital status, alcohol consumption, smoking, BMI (kg/m^2^), hypertension, diabetes, cardiovascular disease (CVD). Finally, a total of 3304 participants were included in this study. The flowchart of this process is shown in Figure 1. This study followed the Strengthening the Reporting of Observational Studies in Epidemiology reporting guideline for cross-sectional studies. All procedures were approved by the NCHS Research Ethics Committee, and participants provided written informed consent.

### 2.3. Exposure variable: CMI

The CMI is calculated based on waist circumference (WC), height, HDL-C, and triglycerides (TG). Height and waist circumference are both expressed in cm, whereas TG and HDL-C are expressed in mg/dL.^[18,19]^ The calculation formula is as follows.


WHtR=WC/height; CMI=(TG/HDL−C)×WHtR.

Briefly, the WHtR was firstly calculated as the ratio of WC to height. Then, CMI was further calculated by multiplying the ratio of TG/HDL-C and WHtR.

### 2.4. Outcome variable: TSH level/primary hypothyroidism

As the dependent variable in this study, the TSH level was measured. Primary hypothyroidism was defined as an TSH level ≥ 4.2 mIU/L.^[20,21]^ Detailed information on the specimen collection, processing, quality assurance, and monitoring are described in the section of biospecimen program in NHANES (www.cdc.gov/nchs/nhanes/).

### 2.5. Covariates

To demonstrate the independent association between the CMI and TSH level/primary hypothyroidism, we performed regression analyses adjusted for potential covariates that might influence this association based on clinical relevance, including sociodemographic factors, health conditions, and lifestyle habits. The selection of covariates was guided by both professional expertise and findings from prior research. The sociodemographic and lifestyle-related variables included in the analysis encompassed gender (male/female), age (years), race (Mexican American, Other Hispanic, Non-Hispanic White, Non-Hispanic Black, Other race), PIR, education level (Less than high school, high school, more than high school), marital status (Never married, Married/Living with partner, Widowed/divorced/Separated), smoking status (categorized as no for participants who smoked < 100 cigarettes in their lifetime and yes for participants who smoked > 100 cigarettes in their lifetime), drink status (categorized as no for participants who drank < 12 alcohol drinks/1 year and yes for participants who drank ≥ 12 alcohol drinks/1 year; Included are liquor (such as whiskey or gin), beer, wine, wine coolers, and any other type of alcoholic beverage).

BMI, height, waist circumference, hypertension, diabetes, CVD, were all regarded as critical indicators of individual health status. Height, waist circumference, and BMI were objectively assessed by trained professionals at the Mobile Examination Center, with BMI computed as the individual’s weight (kg) divided by the square of their height (m^2^). The information regarding hypertension, diabetes and CVD was self-reported by participants via questionnaires. The hypertension was defined based on any of the following criteria: a previous physician diagnosis of hypertension, the average SBP is ≥ 140 mmHg or the average DBP is ≥ 90 mmHg. Diabetes mellitus was defined based on any of the following criteria: fasting plasma glucose ≥ 7 mmol/L, a previous physician diagnosis of diabetes, current use of insulin or other antidiabetic medications. CVD includes any one of congestive heart failure, coronary heart disease, angina/angina pectoris, heart attack, and stroke. Lipid-lowering medication use was defined as self-reported current use of cholesterol-lowering prescription medications (yes/no). Measurements of all study variables can be accessed on the NHANES website.

### 2.6. Statistical analysis

All statistical analyses were conducted in accordance with the guidelines of the CDC, and sampling weights were used to produce nationally representative prevalence estimates for the noninstitutionalized US population. According to the NHANES recommended sample weight on Fasting Subsample 2 Year Mobile Examination Center Weight (WTSAF2YR) records, sample weights of individuals were determined by WTSAF2YR/3. Continuous variables were presented as mean ± standard deviation (SD), while categorical variables were reported as proportions. Analysis of variance and weighted chi-square tests were conducted to assess discrepancies in baseline continuous and categorical variables, respectively. Weighted linear regression and logistic regression modeling were used to investigate the relationship between the CMI and different outcomes (TSH level and primary hypothyroidism). Of the 3 logistic regression models we used, one was unadjusted Model 1. In Model 2, we adjusted for age, gender, race; while in Model 3, we further adjusted for education level, marital status, PIR, CVD, smoking, drinking, diabetes, hypertension, and BMI. To investigate potential non-linear relationships between CMI and TSH level/primary hypothyroidism, we employed restricted cubic splines (RCS) analysis using the “rms” R package (version 6.7.1) with 4 knots. Additionally, subgroup analyses were conducted using logistic regression models stratified by gender (female/male), age (20–60/≥60years), race (Mexican American/Other Hispanic/Non-Hispanic White/Non-Hispanic Black/Other race), education level (less than high school/high school/more than high school), PIR (<1.30/1.30–3.49/≥3.49), marital status (never married/Married or Living with partner/Widowed or divorced or Separated), smoke (yes/no), drink (yes/no), hypertension (yes/no), diabetes (yes/no), CVD (yes/no), BMI (<30/≥30), and lipid-lowering medication use (yes/no). All statistical analyses were performed using R software, with “dplyr” package (version 1.1.4) for data manipulation and preprocessing, “ggplot2” package (version 3.5.0) for plots and visualizations. A 2-sided *P* value < .05 was considered statistically significant.

## 3. Results

### 3.1. Baseline characteristics of the population

Table 1 shows the weighted baseline characteristics of the 3304 participants in this study. The weighted mean age of the participants was 50.37 years, with 50.70% males and 49.30% females. Participants in the highest CMI tertile (T3) group had a higher level of CMI, a higher proportion of population aged 20–60 years, a lower PIR, a higher proportion of males, and a higher proportion of Non-Hispanic whites and individuals who were married/living with a partner compared to those in the lowest CMI tertile (T1). In addition, the highest CMI group had a higher prevalence of hypertension, diabetes, and CVD, a lower level of education compared to the lowest CMI group, and there were some differences in smoking behaviors. Notably, CMI levels were positively associated with primary hypothyroidism prevalence, and the highest CMI tertile group had the highest prevalence of primary hypothyroidism (vs the lowest CMI tertile group, *P* < .001) (Table 1).

**Table 1:** Baseline characteristics of the participants according to the tertiles of the cardiometabolic index (weighted).

		CMI tertiles			
Characteristics	Overall	T1	T2	T3	*P* Value
**Patient numbers no. (%**)	3304	825 (24.97)	1653 (50.03)	826 (24.00)	-
**TSH (mIU/L**)	2.08 ± 1.25	0.98 ± 0.71	1.90 ± 0.74	3.55 ± 1.17	<.001
**Primary hypothyroidism, n (%**)					<.001
Yes	221 (6.69)	18 (2.18)	92 (5.57)	111 (13.44)	
No	3083 (93.31)	807 (97.82)	1561 (94.43)	715 (86.56)	
**Gender, n (%**)					<.001
Male	1675 (50.70)	319 (38.67)	866 (52.39)	490 (59.32)	
Female	1629 (49.30)	506 (61.33)	787 (47.61)	336 (40.68)	
**Age (years**)					<.001
20–60	2147 (64.98)	599 (72.61)	1028 (62.19)	520 (62.95)	
≥60	1157 (35.02)	226 (27.39)	635 (37.81)	306 (37.05)	
**Race, n (%**)					<.001
Mexican American	532 (16.10)	77 (9.33)	282 (17.06)	173 (20.94)	
Other Hispanic	343 (10.38)	53 (6.42)	186 (11.25)	104 (12.59)	
Non-Hispanic white	1598 (48.37)	392 (47.52)	777 (47.01)	429 (51.94)	
Non-Hispanic black	637 (19.28)	246 (29.82)	303 (18.33)	88 (10.65)	
Other race	194 (5.87)	57 (6.91)	105 (6.35)	32 (3.88)	
**PIR**	2.55 ± 1.61	2.70 ± 1.61	2.61 ± 1.61	2.28 ± 1.54	<.001
**Education level, n (%**)					<.001
Less than high school	916 (27.72)	168 (20.36)	451 (27.28)	297 (35.96)	
High school	779 (23.58)	179 (21.70)	407 (24.62)	193 (23.37)	
More than high school	1609 (48.70)	478 (57.94)	795 (48.09)	336 (40.68)	
**Marital status, n (%**)					<.001
Never married	510 (15.44)	182 (22.06)	243 (14.70)	85 (10.29)	
Married/Living with partner	2021 (61.17)	461 (55.88)	1025 (62.01)	535 (64.77)	
Widowed/divorced/Separated	773 (23.39)	182 (22.06)	385 (23.29)	206 (24.94)	
**Drink, n (%**)					.781
Yes	2389 (72.31)	603 (73.09)	1195 (72.29)	591 (71.55)	
No	915 (27.69)	222 (26.91)	458 (27.71)	235 (28.45)	
**Smoking ≥ 100 cigarettes in life, n (%**)					<.001
Yes	1552 (46.97)	315 (38.18)	787 (47.61)	450 (54.48)	
No	1752 (53.03)	510 (61.82)	866 (52.39)	376 (45.52)	
**Hypertension, n (%**)					<.001
Yes	1210 (36.62)	212 (25.70)	596 (36.06)	402 (48.67)	
No	2094 (63.38)	613 (74.30)	1057 (63.94)	424 (51.33)	
**Diabetes, n (%**)					<.001
Yes	434 (13.14)	49 (5.94)	212 (12.83)	173 (20.94)	
No	2870 (86.86)	776 (94.06)	1441 (87.17)	653 (79.06)	
**CVD, n (%**)					<.001
Yes	376 (11.38)	62 (7.52)	189 (11.43)	125 (15.13)	
No	2928 (88.62)	763 (92.48)	1464 (88.57)	701 (84.87)	
**Lipid-lowering medication use, n (%**)					.998
Yes	402 (12.17)	100 (12.12)	200 (12.10)	102 (12.35)	
No	2902 (87.83)	725 (87.88)	1453 (87.90)	724 (87.65)	

CMI = Cardiometabolic Index, CVD = cardiovascular disease, PIR = poverty income ratio, TSH = thyroid-stimulating hormone.

### 3.2. Association between CMI and TSH level/primary hypothyroidism

The weighted linear regression analysis between the CMI and TSH levels showed a positive correlation (Table 2). Prior to regression modeling, collinearity diagnostics were performed, and no significant multicollinearity was detected among the covariates (Supplementary Tables 1–2, Supplemental Digital Content 1). When the CMI was treated as a continuous variable, in Model 1, which did not adjust for any covariates, the β (β = 0.78; 95% confidence interval [CI]: 0.75–0.81, *P* < .001) was statistically significant, indicating that CMI was positively associated with TSH even without accounting for covariates. In Model 3, after adjusting for all covariates, including age, gender, race, education level, marital status, PIR, CVD, smoke, drink, diabetes, hypertension, and BMI, the β coefficient (β = 0.70; 95%CI: 0.66–0.73, *P* < .001) was still statistically significant, meaning that for each unit of the CMI, TSH increased by 0.70 mIU/L. In addition, when the CMI was treated as a categorical variable, in Model 3, after adjusting for all covariates, the β coefficient for the upper 2 tertiles were 2.47 (95%CI: 2.38–2.57) and 0.86 (95%CI: 0.79–0.94) compared with the lowest tertile (*P* for trend < .001).

**Table 2:** Multiple linear regression analysis of the association between CMI and TSH level (weighted).

Variable	Model 1		Model 2		Model 3	
CMI Continuous	β(95% CI)	*P*-value	β(95% CI)	*P*-value	β(95% CI)	*P*-value
	0.78 (0.75–0.81)	<.001	0.77 (0.73–0.79)	<.001	0.70 (0.66–0.73)	<.001
CMI Categories						
T1	0 (reference)		0 (reference)		0 (reference)	
T2	0.91 (0.84–0.98)	<.001	0.90 (0.83–0.98)	<.001	0.86 (0.79–0.94)	<.001
T3	2.57 (2.49–2.65)	<.001	2.55 (2.47–2.64)	<.001	2.47 (2.38–2.57)	<.001
*P* for trend		<.001		<.001		<.001

Model 1: Not adjusted.

Model 2: Adjusted by age, gender, race.

Model 3: Adjusted by age, gender, race, education level, marital status, PIR, CVD, smoke, drink, diabetes, hypertension, BMI, and lipid-lowering medication use.

BMI = body mass index, CI = confidence interval, CMI = Cardiometabolic Index, CVD = cardiovascular disease, PIR = poverty income ratio.

Besides, multiple logistic regression analysis showed that CMI was significantly and positively associated with the prevalence of primary hypothyroidism (Table 3). In Models 3, which adjusted for potential confounders, this correlation remained significant, with a 29% (odds ratio [OR] = 1.29; 95% CI: 1.17–1.42) increase in the risk of primary hypothyroidism prevalence for each unit increase in CMI. In the CMI tertile groupings, the risk of primary hypothyroidism was significantly increased in the highest tertile group (T3) compared with the lowest tertile group (T1). Moreover, the *P*-value of the test for trend showed statistical significance in all models (*P* for trend < .0001), suggesting a progressive increase in the risk of primary hypothyroidism with increasing CMI levels (Table 3).

**Table 3:** Multiple logistic regression analysis of the association between CMI and primary hypothyroidism (weighted).

Variable	Model 1		Model 2		Model 3	
CMI Continuous	OR (95% CI)	*P*-value	OR (95% CI)	*P*-value	OR (95% CI)	*P*-value
	1.31 (1.20–1.43)	<.001	1.30 (1.19–1.42)	<.001	1.29 (1.17–1.42)	<.001
CMI Categories						
T1	Reference		Reference		Reference	
T2	2.64 (1.58–4.41)	<.001	2.79 (1.66–4.68)	<.001	2.96 (1.73–5.05)	<.001
T3	6.96 (4.19–11.57)	<.001	7.47 (4.43–12.61)	<.001	8.70 (4.93–15.37)	<.001
*P* for trend		<.001		<.001		<.001

Model 1: Not adjusted.

Model 2: Adjusted by age, gender, race.

Model 3: Adjusted by age, gender, race, education level, marital status, PIR, CVD, smoke, drink, diabetes, hypertension, BMI and lipid-lowering medication use.

BMI = body mass index, CI = confidence interval, CMI = Cardiometabolic Index, CVD = cardiovascular disease, OR = odds ratio, PIR = poverty income ratio.

### 3.3. Nonlinear analysis of CMI and TSH level/primary hypothyroidism

**Figure 2.**
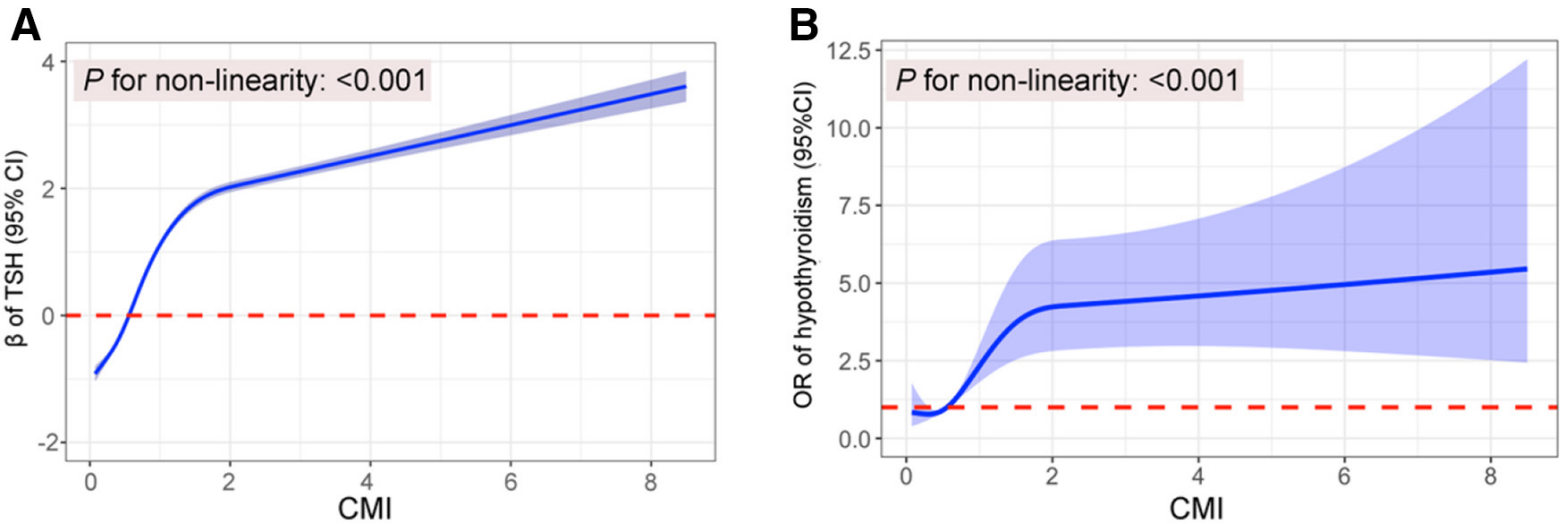
Restricted cubic spline regression curves of the relationships between the CMI and TSH levels (A) and primary hypothyroidism (B) (weighted). TSH = thyroid-stimulating hormone, CMI = Cardiometabolic Index, CI = confidence interval.

Because Model 3 adjusts for all covariates in multiple regression models, it is able to provide estimates that are relatively more stable and closer to the true effect. Therefore, we used RCS analysis based on this model to explore whether there was a nonlinear relationship between CMI and TSH. The findings, which are displayed in Figure 2A, demonstrate that there is a nonlinear relationship between CMI and TSH level. The *P* value for nonlinearity was < 0.001. After multivariate adjustment, the inflection point of the association between CMI and TSH level was found to be 0.55. Besides, after multivariate adjustment, a nonlinear relationship was found in CMI and primary hypothyroidism, the inflection point of the association between CMI and primary hypothyroidism was found to be 0.56, the *P* value for nonlinearity was < .001 (Fig. 2B).

### 3.4. Subgroup analyses

**Figure 3.**
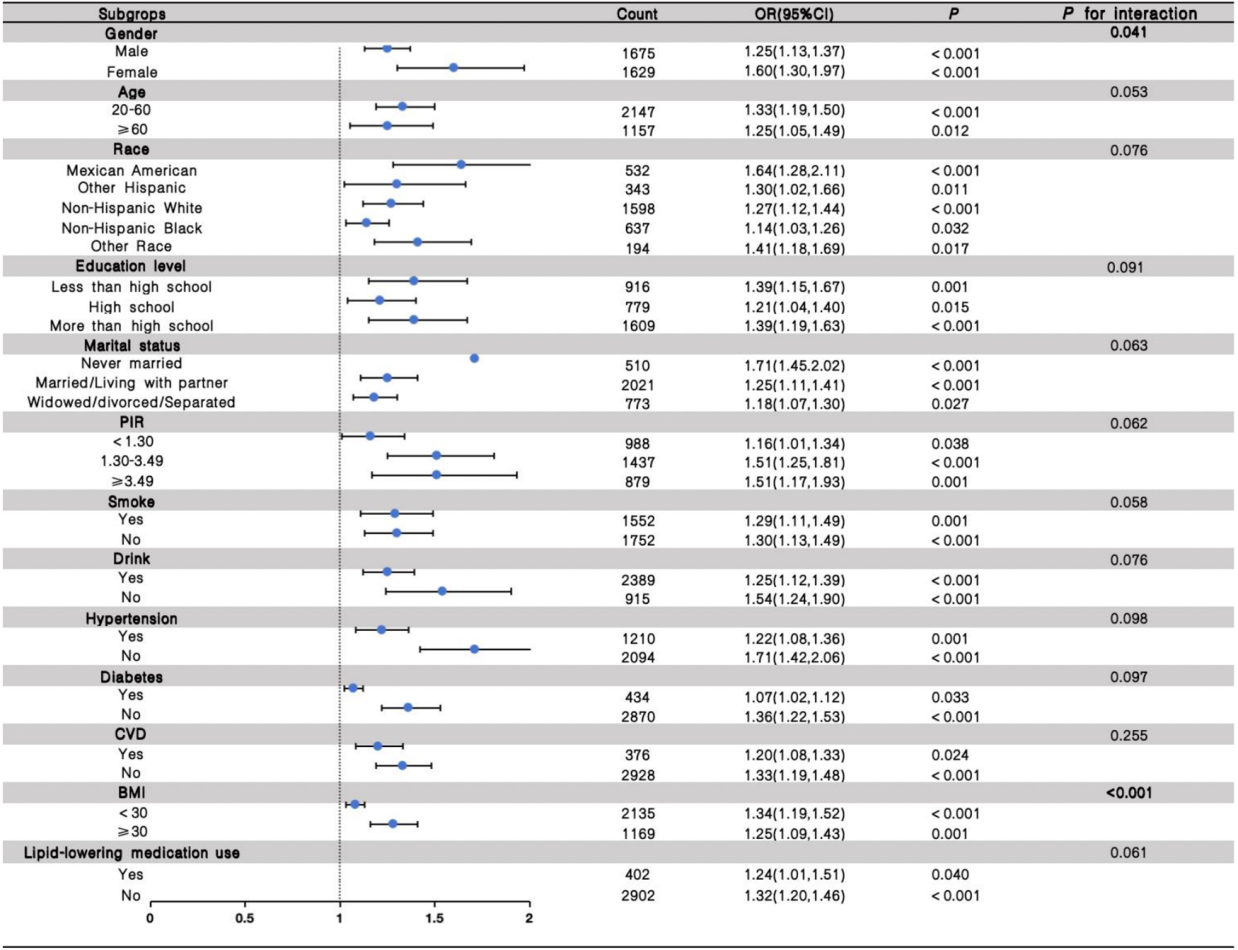
The association between the CMI and primary hypothyroidism by selected subgroups (weighted). PIR = poverty income ratio, CVD = cardiovascular disease, BMI = body mass index.

In order to assess the consistency of the relationship between CMI and primary hypothyroidism across different subgroups, this study conducted subgroup analyses (Fig. 3). Subgroup analysis stratified by gender (female/male), age (20–60/≥60 years), race (Mexican American/Other Hispanic/Non-Hispanic White/Non-Hispanic Black/Other race), education level (less than high school/high school/more than high school), PIR (<1.30/1.30–3.49/≥3.49), marital status (never married/Married or Living with partner/Widowed or divorced or separated), smoke (yes/no), drink (yes/no), hypertension (yes/no), diabetes (yes/no), CVD (yes/no) BMI (<30/≥30), and the use of cholesterol-lowering prescription medications (yes/no). The results showed that the association of the CMI with primary hypothyroidism was stable present in all subgroups. Notably, no significant interactions were observed in the age, ace, education level, PIR, marital status, smoke, drink, hypertension, diabetes, and CVD, indicating that the association did not depend on these variables (*P* for interaction > .05). However, the gender and BMI were found to significantly influence the strength of their association (*P* for interaction < .05).

## 4. Discussion

To our knowledge, this is the first population-based study to investigate the relationship between CMI level and TSH level/primary hypothyroidism risk. Our study revealed that CMI level was an independent risk factor for primary hypothyroidism risk using multivariate-adjusted regression analysis, which remained significant after adjusting for all potential confounders. Moreover, we observed a dose-dependent association between CMI and TSH level/primary hypothyroidism risk in US adults.

As a highly sensitive indicator of thyroid function, TSH is capable of reflecting both hypothyroidism and subclinical hyperthyroidism.^[22]^ Studies have shown that TSH is closely associated with lipid metabolism, and the degree of lipid derangement is generally proportional to the degree of TSH elevation.^[23–25]^ Several studies consistently demonstrate that even when TSH levels remain within the clinical reference range, an elevation in TSH is accompanied by corresponding increases in serum total cholesterol (TC), low-density lipoprotein cholesterol (LDL-C), TG, as well as a reduction in HDL-C.^[26–29]^ Additionally, thyroid hormone treatment is efficacious in reversing the lipid abnormalities in both subclinical and overt hypothyroidism. In 2015, Ichiro Wakabayashi proposed the CMI-a novel indicator designed to assess central obesity.^[30]^ Integrating parameters related to abdominal obesity and lipid metabolism, CMI has been shown to correlate with a range of metabolic disorders; additionally, it exhibits predictive value for the risk of obesity- and dyslipidemia-induced conditions, including diabetes mellitus, coronary artery disease, and metabolic syndrome.^[31]^ In our study, we found a close correlation between CMI levels and primary hypothyroidism, and this correlation becomes more pronounced when CMI exceeds 0.59.

The mechanisms underlying the close association between the CMI and primary hypothyroidism may be multifaceted. First, one of the core pathological bases for elevated CMI is IR,^[32]^ which is often accompanied by increased waist circumference, hyperglycemia, and hypertriglyceridemia. In turn, IR can disrupt the balance of thyroid function through a “central-peripheral” dual pathway.^[33]^ On one hand, in the state of IR, thyroid hormone synthesis may be reduced; this weakens the negative feedback inhibition on the hypothalamic-pituitary-thyroid axis at the hypothalamic-pituitary level, thereby upregulating the secretion of TSH. On the other hand, during IR, the weakened insulin signalingcan lead to a “decreased affinity” of the thyroid-stimulating hormone receptor for TSH, triggering a compensatory increase in TSH.^[34]^ In addition, elevated CMI is often accompanied by chronic low-grade inflammation (e.g., proinflammatory cytokines secreted by visceral adipose tissue, vascular endothelial inflammation induced by hyperglycemia), and inflammation may serve as a key “mediating factor” linking CMI to elevated TSH and primary hypothyroidism.^[35]^ Under the inflammatory state associated with CMI, adipose tissue (especially visceral adipose tissue) and immune cells secrete large amounts of proinflammatory cytokines (e.g., TNF-α, IL-6, IL-1β). After these cytokines reach the thyroid gland via the bloodstream, they can inhibit the activity of the sodium-iodide symporter in thyroid follicular epithelial cells, leading to insufficient iodine uptake.^[36]^ This directly reduces thyroid hormone synthesis and further affects TSH levels through negative feedback. Besides, proinflammatory cytokines (e.g., IL-6) may also affect the glycosylation modification of TSH, leading to a “decreased proportion of active TSH.” As a result, the thyroid gland is unable to synthesize hormones normally, forming a vicious cycle of “pseudo-elevation of TSH + thyroid functional insufficiency.” Besides, elevated CMI, particularly in the context of obesity, is often accompanied by “leptin resistance.” Long-term leptin resistance can reduce the sensitivity of thyroid cells to TSH, resulting in a compensatory increase in TSH. Conversely, high levels of TSH can further affect lipid metabolism.

In conclusion, this study has certain research strengths. First, this study is the first to explore the correlation between CMI and primary hypothyroidism using cross-sectional data, which provides a direction for future research. Secondly, this study is based on the NHANES database, which has a large and nationally representative sample size and provides reliable data support for the results. Thirdly, this study corrected for a variety of potential confounders through extensive questionnaires and multivariate modeling to ensure the accuracy and credibility of the findings. Fourth, the measurement of CMI is simple, cost-effective, and has good clinical applicability, which facilitates the assessment and screening of thyroid-related diseases. This study validates the feasibility of CMI as a potential marker in the assessment of primary hypothyroidism risk and prevalence and lays the foundation for its application as a risk prediction tool in clinical practice, which holds substantial importance for the advancement of strategies concerning the prevention and early intervention for primary hypothyroidism.

Nevertheless, there are several restrictions on the current study. First, it is important to acknowledge that a limitation of cross-sectional studies collect data at a single point in time, this study cannot determine whether CMI precedes primary hypothyroidism or vice versa. The results may be influenced by this temporal relationship uncertainty and thus require cautious interpretation. Secondly, the data related to thyroid function used in this study did not distinguish between clinical primary hypothyroidism and subclinical primary hypothyroidism, thus overlooking differences in their etiological classifications and other aspects. Thirdly, although we adjusted for a variety of possible confounders in our model, there may still be unadjusted residual confounders that affect the accuracy of the study findings. Furthermore, due to the limitations in the included population, the relatively small sample sizes in certain subgroups may restrict the statistical power of subgroup analysis and interaction tests. This can potentially affect the results of the regression analysis, increasing the instability of the findings. Hence, for a more in-depth examination of the relationship between CMI and primary hypothyroidism, future research endeavors should concentrate on extensive cohort studies that monitor the fluctuating trends of CMI longitudinally and explore its potential temporal correlation with the inception and progression of primary hypothyroidism. Moreover, future studies should explore the complex biological mechanisms that regulate the interaction between CMI and primary hypothyroidism. By addressing these challenges and leveraging these opportunities, it is anticipated that future research will yield more comprehensive and in-depth insights-thereby driving substantial progress in investigating the relationship between CMI and primary hypothyroidism.

## 5. Conclusions

The study found that CMI levels are closely correlated with primary hypothyroidism, with this correlation increasing when the CMI exceeds 0.59. This observation suggests that routine monitoring of CMI levels could equip clinicians to identify individuals at heightened risk of primary hypothyroidism at an earlier stage. Such early detection, in turn, would facilitate the timely initiation of targeted interventions, which may help mitigate the progression of primary hypothyroidism and its associated health impacts. However, to strengthen the robustness and generalizability of these preliminary findings, additional large-scale prospective cohort studies-preferably with long-term follow-up to track incident primary hypothyroidism cases-are warranted to replicate and validate the observed associations between CMI and primary hypothyroidism risk.

## Acknowledgments

Thank the Fifth Clinical Medical College of Henan University of Traditional Chinese Medicine for their assistance with manuscript submission.

## Author contributions

**Conceptualization:** Xue-kang Ren, Yu Fu, Shao-gong Zhu, Yun-zhan Xia.

**Data curation:** Xue-kang Ren, Yu Fu, Shao-gong Zhu, Rong-zhen Li, Yun-zhan Xia.

**Formal analysis:** Yu Fu, Shao-gong Zhu.

**Investigation:** Xue-kang Ren, Yu Fu, Shao-gong Zhu, Rong-zhen Li.

**Methodology:** Xue-kang Ren, Yu Fu, Rong-zhen Li.

**Supervision:** Yun-zhan Xia.




