# Supplementary material for: Association between cardiometabolic index and primary hypothyroidism: A cross-sectional study based the NHANES (2007–2012)
Source: Medicine (Baltimore). 2026 Jun 12;105(24):e49270. doi: 10.1097/MD.0000000000049270 (PMC13268431; doi:10.1097/MD.0000000000049270)
Supplement: Supplementary file 2 [file medi-105-e49270-s002.docx]

## Supplementary Table2. Collinearity Diagnostics (CMI as a categorical variable)

| Variables | VIF |
| --- | --- |
| CMI(T2) | 1.7 |
| CMI(T3) | 2.0 |
| gender | 1.1 |
| age | 1.6 |
| race | 1.1 |
| education level | 1.4 |
| marital status | 1.2 |
| PIR | 1.4 |
| BMI | 1.2 |
| CVD | 1.6 |
| smoking status | 1.1 |
| drink | 1.1 |
| hypertension | 1.3 |
| diabetes | 1.1 |
| lipid-lowering medication use | 1.5 |

CMI=Cardiometabolic Index, PIR= Poverty Income Ratio, CVD=Cardiovascular disease, BMI= Body Mass Index.
